# Targeting the Epidermal Growth Factor Receptor in EGFR-Mutated Lung Cancer: Current and Emerging Therapies

**DOI:** 10.3390/cancers13133164

**Published:** 2021-06-24

**Authors:** Karam Khaddour, Sushma Jonna, Alexander Deneka, Jyoti D. Patel, Mohamed E. Abazeed, Erica Golemis, Hossein Borghaei, Yanis Boumber

**Affiliations:** 1 Division of Hematology and Oncology, University of Illinois at Chicago, Chicago, IL 60612, USA; sjonna@uic.edu; 2 Fox Chase Cancer Center, Program in Molecular Therapeutics, Philadelphia, PA 19111, USA; alexander.deneka@fccc.edu (A.D.); erica.golemis@fccc.edu (E.G.); 3 Robert H. Lurie Comprehensive Cancer Center, Division of Hematology/Oncology, Feinberg School of Medicine, Northwestern University, Chicago, IL 60611, USA; jd-patel@northwestern.edu; 4 Robert H. Lurie Comprehensive Cancer Center, Department of Radiation Oncology, Feinberg School of Medicine, Northwestern University, Chicago, IL 60611, USA; mabazeed@northwestern.edu; 5 Fox Chase Cancer Center, Department of Hematology and Oncology, Philadelphia, PA 19111, USA; hossein.borghaei@fccc.edu; 6 Institute of Fundamental Medicine and Biology, Kazan Federal University, 420008 Kazan, Russia

**Keywords:** lung cancer, adenocarcinoma, overall survival, EGFR, tyrosine kinase inhibitors, progression-free survival

## Abstract

**Simple Summary:**

Epidermal growth factor receptor (EGFR) mutations occur in a significant number of lung cancer patients. Treatment outcomes in this subset of patients has greatly improved over the last decade after the introduction of EGFR tyrosine kinase inhibitors (TKIs), which demonstrated high efficacy and improved survival in randomized clinical trials. Although EGFR TKIs became the standard of care in patients with EGFR-mutated lung cancer, resistance almost inevitably develops. This constitutes a major challenge and creates an unmet need for novel therapies and new approaches to ameliorate or overcome this resistance. In this review we discuss currently approved TKIs for the targeted management of EGFR-mutated lung cancer. We also review common mechanisms of resistance to EGFR-targeted therapies and describe promising approaches that may mitigate resistance.

**Abstract:**

Epidermal growth factor receptor-targeting tyrosine kinase inhibitors (EGFR TKIs) are the standard of care for patients with EGFR-mutated metastatic lung cancer. While EGFR TKIs have initially high response rates, inherent and acquired resistance constitute a major challenge to the longitudinal treatment. Ongoing work is aimed at understanding the molecular basis of these resistance mechanisms, with exciting new studies evaluating novel agents and combination therapies to improve control of tumors with all forms of EGFR mutation. In this review, we first provide a discussion of EGFR-mutated lung cancer and the efficacy of available EGFR TKIs in the clinical setting against both common and rare EGFR mutations. Second, we discuss common resistance mechanisms that lead to therapy failure during treatment with EGFR TKIs. Third, we review novel approaches aimed at improving outcomes and overcoming resistance to EGFR TKIs. Finally, we highlight recent breakthroughs in the use of EGFR TKIs in non-metastatic EGFR-mutated lung cancer.

## 1. Introduction

**Figure 1:**
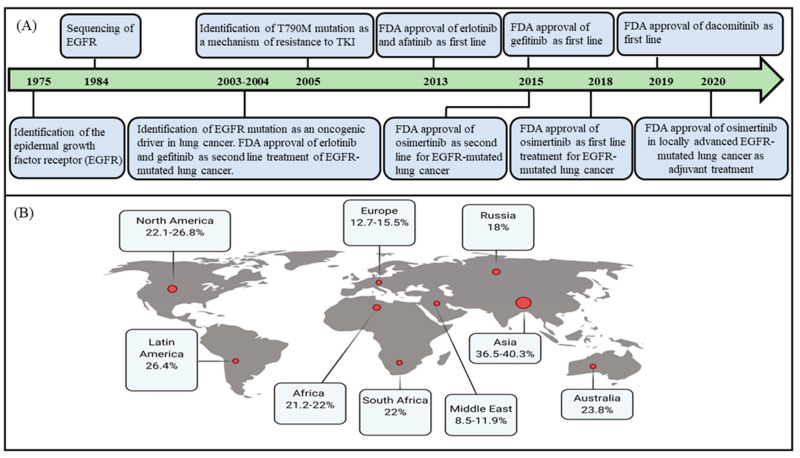
EGFR-mutated lung adenocarcinoma. Panel (**A**): A timeline of major developments in EGFR-mutated lung adenocarcinoma. Panel (B): The reported frequencies of EGFR mutations in lung adenocarcinoma based on geographic region: frequency in North America [29,30,31], Asia [27,28,29], Russia [32], South Africa [33], Middle East and Africa [34], Australia [35], and Latin America [36].

Targeting activating mutations in the tyrosine kinase epidermal growth factor receptor (EGFR) represents a success story in the treatment of lung cancer, a very commonly diagnosed malignancy that remains the leading cause of cancer-related mortality (Figure 1A) [1]. Tyrosine kinase inhibitors for EGFR (EGFR TKIs) were initially developed to target tumors overexpressing EGFR, to counteract the resulting kinase overactivation [2,3]. At the time of development of EGFR TKIs, it was unknown that some lung cancers bore activating mutations in EGFR. Despite the fact that initial clinical trials showed higher objective response rates (ORR) with EGFR TKIs compared to chemotherapy in lung cancer (specifically non-small cell lung cancer, NSCLC), these trials did not establish an advantage in the general population for progression-free survival (PFS) or overall survival (OS), as these trials enrolled lung cancer patients irrespective of EGFR mutation status [4,5,6]. Subsequent studies analyzing molecular alterations in the tumors of lung cancer patients who had an excellent response to EGFR TKIs revealed the presence of unique mutations in EGFR that conferred high sensitivity to EGFR TKIs [7,8,9]. In a first study, Lynch et al. sequenced the codon region of EGFR in nine lung adenocarcinoma patients who had a major or partial response to gefitinib (a first-generation EGFR TKI), and identified somatic mutations in the EGFR kinase domain [7]. The majority of EGFR mutations were either in-frame deletions of exon 19 or single amino acid substitutions (L858R) in exon 21 and were later termed as sensitizing mutations. Subsequent studies using various EGFR TKIs similarly demonstrated higher ORR and longer PFS compared to platinum-based doublet chemotherapy both in recurrent metastatic lung adenocarcinoma and in frontline treatment of lung adenocarcinoma patients with sensitizing EGFR mutations (in this review we will be using the term lung adenocarcinoma to refer to non-small cell lung cancer with adenocarcinoma histology) [10,11,12,13,14,15,16,17,18,19,20,21,22,23,24,25,26].

Unexpectedly, the initial trials of EGFR TKIs identified higher ORR and improved outcomes among young women, non-smokers, and Asians with lung cancer [7,8], reflecting the fact that the frequency of EGFR mutations is higher in these patient groups. Ethnicity appears to strongly influence the incidence of detected EGFR mutations, which is reflected by the variable distribution of such mutations in regions with demographic profiles reflecting distinct ancestries (Figure 1B).

**Figure 2:**
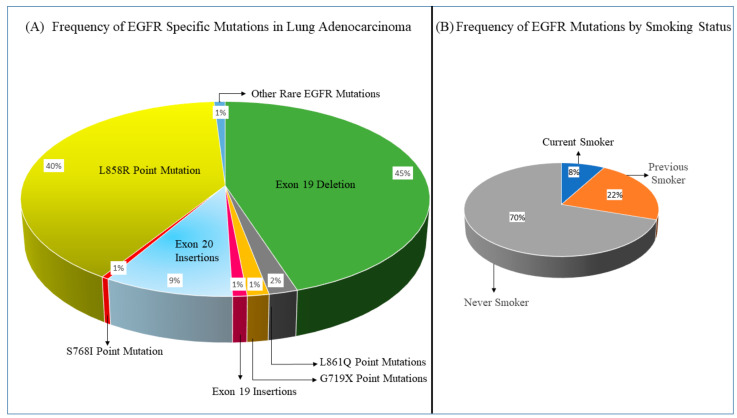
Frequency of EGFR mutations in lung adenocarcinoma. Panel (**A**): Common and rare EGFR mutations and their frequencies in lung adenocarcinoma [37]. Panel (**B**): EGFR mutation frequency based on smoking status [37].

The highest prevalence of EGFR mutations (30–38%) occurs in lung adenocarcinoma patients of Southeast Asian ethnicity, including Japan and China [27,28,29]. In contrast, Caucasians have a lower mutation prevalence of 16–17% [29,30,31]. Similarly, ethnic-epidemiological studies have identified different frequencies of EGFR mutations in Russia, South Africa, the Middle East and Africa, Australia, and Latin America (18%, 23%, 21.2%, 23.8%, and 26%, respectively) [32,33,34,35,36]. EGFR mutation prevalence can also vary depending on smoking status, sex, and lung cancer histology. For example, the genotyping of 3026 lung adenocarcinoma samples revealed a higher frequency of EGFR mutations in never smokers (42.5%) and former smokers (13.5%) compared to current smokers (4.9%); the most common EGFR mutations in this study included exon 19 deletions and L858R point mutations (Figure 2A,B) [37]. It has been suggested that the higher incidence of EGFR mutations in women compared to men reflects the higher proportion of nonsmokers among females [38]. Moreover, the prevalence of EGFR is highest among patients with NSCLC with an adenocarcinoma histology, while it occurs only rarely in NSCLC patients with squamous cell carcinoma histology and small cell lung cancer (SCLC) [39,40,41].

The initial approval of EGFR TKIs as standard therapies for patients with specific EGFR mutations (mainly exon 19 deletions and L858R point mutations) was based on the results of randomized clinical trials that demonstrated the superiority of this class of treatment agents versus platinum-based doublet chemotherapy in lung adenocarcinoma (Table 1). Two first-generation EGFR TKIs, erlotinib and gefitinib, demonstrated a significantly longer median PFS compared to platinum-based chemotherapy as front-line treatment in patients with metastatic lung cancer harboring sensitizing EGFR mutations [11,12,13,14,15,16,17,18,19].

EGFR is one of four members of the ERBB protein family, which also includes HER2, ERBB3, and ERBB4. The pan-ERBB inhibitor, afatinib, is a second-generation TKI which also demonstrated longer PFS in metastatic EGFR-mutated lung adenocarcinoma compared to standard chemotherapy [20,21,22]. Another second-generation EGFR TKI, dacomitinib, was assessed in the ARCHER 1050 trial. In this study, treatment with dacomitinib was associated with a longer median PFS than was seen for gefitinib (mPFS 14.7 versus 9.2 months, respectively), albeit higher drug-related adverse events [23,24]. Collectively, these trials established EGFR TKIs as front-line treatments in locally advanced and metastatic (stage IV) lung adenocarcinoma with sensitizing EGFR mutations.

Despite the promising activity of EGFR TKIs, intrinsic or acquired resistance mechanisms limited their efficacy. For example, emergence of a tumor bearing an EGFR T790M mutation in a patient who had disease progression after initial response to gefitinib was first identified in 2005 [42]. Subsequent research established T790M mutation as the main (“gatekeeper”) mutation conferring resistance to first- and second-generation EGFR TKIs, occurring at an estimated frequency of 50–70% in tumors progressing while on EGFR TKI therapy [43,44,45]. However, T790M has also been detected in tumors previously untreated with EGFR TKs, suggesting an additional role in primary resistance to first- and second-generation TKI [43]. The high prevalence of T790M mutation motivated a search for third-generation EGFR TKIs that are active in the context of this resistant mutation. Among these is osimertinib, which has a high affinity for binding to the ATP pocket in the kinase domain of EGFR and, correspondingly, has excellent activity against T790M-mutated lung adenocarcinoma. The phase-2 AURA2 trial assessing the safety and efficacy of osimertinib in TKI-pretreated patients with EGFR-mutated lung adenocarcinoma bearing T790M mutations demonstrated a 70% ORR [46]. Subsequently, the phase 3 FLAURA study compared osimertinib to either erlotinib or gefitinib in frontline therapy for EGFR-mutated lung adenocarcinoma. In this trial, osimertinib was associated with a longer mPFS compared to first-generation EGFR TKIs (18.9 months vs. 10.2 months, *p* < 0.001) [25]. An updated survival analysis from the FLAURA trial demonstrated prolonged OS in the osimertinib arm compared to patients who received earlier generation TKI (38.6 months vs. 31.8 months, *p* = 0.046 respectively) [26]. Moreover, osimertinib demonstrated higher efficacy in controlling intracranial metastatic disease and lower CNS progression rate when compared to first-generation EGFR TKI (6% vs. 15% intracranial progression rate respectively) [25].

Despite the substantial improvement in the treatment of EGFR-mutated lung adenocarcinoma, there remain key issues for clinical practice that motivate ongoing research. Herein, we discuss the most common sensitizing EGFR mutations and their clinical relevance to treatment choice. We also discuss the evidence as to the efficacy of EGFR TKIs in tumors bearing less common EGFR mutations. Next, we discuss some important resistance mechanisms implicated in TKI failure. We provide a discussion of available combination and novel therapies that are being studied to improve outcomes and target resistant mechanisms in EGFR-mutated lung cancer. Finally, we highlight the recent breakthrough in the use of EGFR TKIs in non-metastatic lung adenocarcinoma.

## 2. Current Treatment Landscape of EGFR-Mutated Lung Adenocarcinoma 

**Figure 3:**
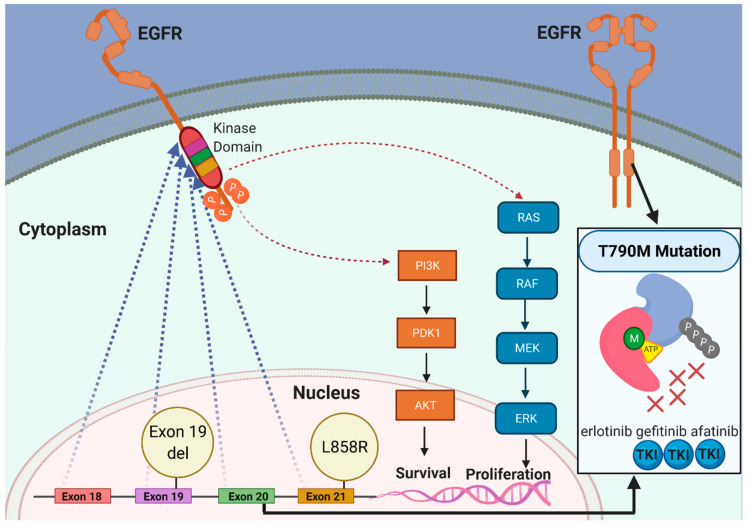
The biology of EGFR mutations in lung cancer. In normal cells, binding of a ligand causes dimerization of EGFR which leads to downstream activation of several pathways such as PI3K/AKT and MAPK/RAF which control cell growth and proliferation. In EGFR-mutated lung cancer, activating somatic mutations in exons 18–21 can lead to constant activation of the EGFR kinase domain with continuous downstream signaling through PI3K/AKT and MAPK/RAF pathways irrespective of ligand binding to growth hormones. This can lead to cell survival, proliferation and resistance to apoptosis. The most common EGFR mutations are exon 19 deletions and a point mutation in exon 21 (L858R). The presence of the point mutation T790M in exon 20 leads to resistance to first- and second-generation EGFR TKIs due to a higher affinity of the ATP-binding pocket in the EGFR to ATP and a lower affinity to first- and second-generation EGFR TKIs such as erlotinib, gefitinib, afatinib, and dacomitinib.

The majority of EGFR mutations affect the intracellular tyrosine kinase domain of the receptor, and encompass a heterogeneous group of mutations in exons 18 to 21 (Figure 3). Activating somatic EGFR mutations, which are considered oncogenic in lung cancer, can be divided into common mutations with known sensitizing activity to TKIs, and uncommon mutations. The common mutations constitute more than 85% of EGFR somatic mutations in lung adenocarcinoma. These include in-frame deletions in exon 19 (45%) and a missense mutation in exon 21 (L858R) (40%) [47]. Exon 19 deletions and L858R mutations lead to an enhanced tyrosine kinase activity and increased downstream signaling due to constant dimerization of EGFR which is responsible for its oncogenic potential [48]. These common mutations are highly sensitive to first-, second-, and third-generation EGFR TKIs, and are thereby referred to as sensitizing mutations. Less frequent EGFR mutations constitute the remaining 10–15% of EGFR-mutated lung adenocarcinoma, and comprise a heterogeneous group of rare mutations with the most common being reported in exon 18 (E709X and G719X), exon 19 (exon 19 insertions), exon 20 (exon 20 insertions and S768I), and exon 21 (L861Q). Importantly, different mutations can occur simultaneously and are referred to as compound or complex mutations; these have been reported in up to 15% of EGFR-mutated lung adenocarcinoma [49].

The most clinically relevant EGFR TKI resistance mutation is the T790M missense mutation, which occurs in exon 20. The clinical importance of T790M mutation stems from its high frequency in EGFR-mutated lung adenocarcinoma at progression during treatment with first- and second-generation EGFR TKI (50–70%) [43,44,45]. This mutation increases ATP affinity at the binding pocket of the receptor and prevents first- and second-generation EGFR TKIs from binding to the ATP region [50]. Lung adenocarcinomas bearing T790M mutations are notoriously resistant to first-generation TKI. Although lung adenocarcinoma cells bearing this mutation are sensitive to high concentrations of second-generation EGFR TKIs in vitro, concentrations effective in controlling growth of these tumors in vivo are too toxic to be used in humans [51]. Therefore, the current standard of care in the frontline setting in many countries (including the US, Japan, and the EU) is osimertinib, due to its high efficacy against T790M mutations; however, in some countries, osimertinib is only available for patients with T790M mutations that have arisen after progressing during treatment with first- or second-generation EGFR TKIs.

### 2.1. EGFR TKIs against EGFR-Mutated Lung Adenocarcinoma with Common Sensitizing EGFR Mutations

As the majority of patients in the TKI clinical trials in EGFR-mutated lung adenocarcinoma were younger than the general population of lung cancer patients and were selected for good performance status, it has been important to establish safety and efficacy in a real-world setting. After a number of years of use of these drugs, it is clear that EGFR TKIs are effective in controlling EGFR-mutated lung adenocarcinoma, and have a favorable safety and tolerability profile, including in elderly patients. For example, in a multicenter study, Corre et al. demonstrated comparable efficacy and tolerability of first- and second-generation EGFR TKIs in EGFR-mutated lung adenocarcinoma patients >80 years old (ORR 63.3% and mPFS of 11.9 months) [52]. Another large study in Japan demonstrated non-inferior outcomes for safety profile and mPFS in elderly patients with EGFR-mutated lung adenocarcinoma (age > 75 years old) compared to relatively younger patients who were treated with erlotinib [53].

Real world evidence has been recently reported for osimertinib, with outcomes which replicated clinical trial outcomes [54,55,56]. As one example, a global real-world study (ASTRIS) of 3015 patients with EGFR T790M lung adenocarcinoma who received osimertinib after progression to EGFR TKIs found an ORR of 75.1% and mPFS of 11.1 months, with a toxicity profile comparable to the FLAURA study [54]. A retrospective French study of 205 patients (mean age 69.5) who received osimertinib as a second- or third-line therapy after progression on earlier generation EGFR TKIs found PFS and OS similar to those observed in clinical trials of osimertinib [55]. Similarly, a Chinese retrospective study of 74 EGFR-mutated lung adenocarcinoma patients who received at least three prior lines of therapy and were later treated with osimertinib found comparable ORR and PFS to randomized clinical trials [56]. Of interest, a recent analysis of 31 patients who were treated with osimertinib suggested a longer mPFS in elderly patients (≥65 years) compared to younger patients (6.4 months vs. 3.5 months, *p* = 0.41 respectively), albeit conclusions of this study are limited by small sample size [57]. Collectively, real-world evidence has yielded similar outcomes to osimertinib clinical trials, causing this agent to be considered the standard of care in the general population including elderly patients with EGFR-mutated lung adenocarcinoma [58].

The superior tolerability of EGFR TKIs compared to chemotherapy in lung cancer patients has been demonstrated consistently in the randomized clinical trials noted in Table 1, and the use of EGFR TKIs was described in some studies to be associated with improved quality of life [59,60]. Similarly supporting quality of life, the ability to administer EGFR TKIs orally yields additional benefits to physicians and patients, including reduction of travel during treatment.

**Figure 4:**
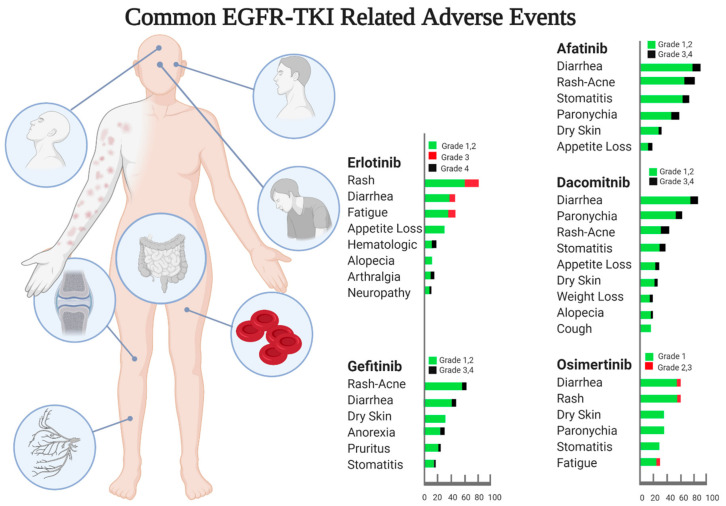
Common reported adverse events from phase 3 randomized clinical trials of EGFR TKIs in EGFR-mutated lung cancer. These include first-generation: erlotinib [13], gefitinib [19], second-generation including afatinib [20], dacomitinib [23], and third-generation osimertinib [25] EGFR TKI drugs.

The most common adverse events (AEs) reported in the clinical trials of EGFR TKIs are cutaneous and gastrointestinal (GI) toxicities (Figure 4). Mucocutaneous toxicities caused by EGFR TKIs can include acne-like eruption, folliculitis, xerosis, paronychia, mucositis, and stomatitis, which are caused by disrupted function of keratinocyte in the epidermis [61]. Of interest, several reports including a recent meta-analysis suggested a correlation between the development of skin toxicities and higher response rates to early generation EGFR TKIs [62]. GI toxicity is typically caused by the irritation of the GI mucosa. In addition, other AEs occurring at lower frequencies, have been reported to be associated with use of EGFR TKIs: these include appetite loss, hematologic abnormalities, arthralgia, fatigue, and cough (Figure 4). Notably, osimertinib has the advantage of causing less toxicities and is more well-tolerated compared to first- and second-generation EGFR TKIs owing to its high selectivity to bind mutated EGFR versus wild-type EGFR.

The majority of AEs associated with use of EGFR TKIs are mild to moderate. Management of EGFR TKI- associated AEs is based on dose modification, supportive care, and brief medication discontinuation until resolution of toxicity [63,64]; the need for permanent discontinuation of EGFR TKIs due to AEs is uncommon.

### 2.2. Efficacy of EGFR TKIs in Lung Adenocarcinoma with Rare EGFR Mutations and Exon 19 and 20 Insertions

Data on the sensitivity of infrequent EGFR mutations (that is, excluding exon 19 deletions and L858R mutations) to currently approved EGFR TKIs varies, and have been reported mostly in retrospective studies and case reports (Table 2). Interestingly, some of the uncommon mutations coexist with exon 19 deletion or L858R mutations; patients with these complex EGFR mutations may have greater sensitivity and achieve longer remission following treatment EGFR TKIs, compared to patients who harbor noncomplex sensitizing mutations [65].

The G719X mutation (where X reflects several potential missense substitutions) in exon 18 constitutes approximately 1.5–3% of all EGFR mutations in lung adenocarcinoma [66]. Chiu et al. demonstrated ORR of 36.8% and mPFS of 6.3 months in patients with lung tumors bearing a G719X mutation, and treated with erlotinib or gefitinib [67]. The presence of exon 19 deletions or co-occurrence of L858R and G719X mutations was associated with higher response rates and longer PFS compared to G719X mutations alone [66]. Afatinib demonstrated activity against G719X mutations in a pooled analysis from three randomized controlled trials of afatinib (LUX-Lung 2, LUX-Lung 3 and LUX-Lung 6) [68]. Similarly, osimertinib has demonstrated activity against G719X in 19 patients with ORR of 52.6%; mPFS had not been reached at the time of publication [69]. 

Insertions in exon 19 are uncommon and account for only 1% of all EGFR mutations [70]. First-generation EGFR TKI including erlotinib and gefitinib appear to have good efficacy against exon 19 insertions, with an ORR of 40% (albeit in a very small study of 10 patients) [66]. Afatinib achieved partial responses in a case series of 12 patients with EGFR-mutated lung adenocarcinoma who had exon 19 insertion [71]. 

In addition to the dominant resistance-associated mutation T790M, there are other low frequency mutations in exon 20. A heterogeneous group of amino acid insertions in exon 20 have been reported, with such insertions constituting 10% of EGFR-mutated lung adenocarcinoma [72]. The efficacy of first-generation EGFR TKIs against exon 20 insertions is very limited (ORR 8–27% and mPFS 2–2.5 months), and chemotherapy remains the standard of care in these patients [73,74]. For example, in a French multicenter observational study the ORR in patients with exon 20 insertions who were treated with either erlotinib or gefitinib was 8% (*n* = 38), with mPFS of 2 months [73]. Another study by Naidoo et al. exploring use of erlotinib in patients with exon 20 insertions reported an ORR of 27% (3 of 11 patients) and mPFS of 2.5 months [74]. Afatinib had limited efficacy in lung cancer patients with exon 20 insertions, based on a pooled analysis by Yang et al., which found an ORR of 8.7% and mPFS of 2.7 months [68]; similarly, dacomitinib showed modest efficacy in a study of 6 patients (1 partial response, 2 stable disease, and 2 progressive disease) [75]. Osimertinib showed also mild activity against exon 20 insertions in a phase II trial that evaluated a dose of 160 mg and found an ORR of 25% with mPFS of 9.7 months [76]. The relative high frequency of exon 20 insertions compared to other uncommon mutations and the lack of good response to approved TKIs has resulted in an effort to investigate novel therapeutics to overcome this resistant alteration in EGFR-mutated lung adenocarcinoma, discussed below. S768I is another relatively uncommon mutation in exon 20 that accounts for 0.5% of EGFR-mutated lung cancer [77]. Among EGFR TKIs, afatinib showed the best efficacy with ORR of 100% and mPFS of 14.7 months, albeit in a very small study of eight patients [68,69,77]. Finally, the L861Q mutation, occurring in exon 21 with 3% frequency, is considered to be sensitive to afatinib and osimertinib (ORR 56.3% and 77.8%, respectively) [66,68,69,78]. 

### 2.3. Treatment of Brain Metastases in EGFR-Mutated Lung Adenocarcinoma Using EGFR TKIs 

At least one-third of patients with EGFR-mutated lung adenocarcinoma develop brain metastases during the course of the disease [79]. The incidence of brain metastases is elevated in EGFR-mutated lung adenocarcinoma, with a frequency of associated intracranial disease reaching 31% in EGFR-mutated lung adenocarcinoma compared to 19.7% in EGFR-wild type lung adenocarcinoma [80,81]. The presence of brain metastases is associated with increased morbidity and shortened OS [82]. Despite the improved outcomes with EGFR TKIs, most clinical trials excluded patients with intracranial disease or had small number of patients with brain metastasis. However, trials of osimertinib were allowed to enroll patients with treated brain metastasis and have confirmed the superiority of osimertinib CNS efficacy over first-generation EGFR TKIs [46]. The FLAURA trial enrolled 128 EGFR-mutated lung adenocarcinoma patients with brain metastases, and reported that median CNS PFS that was not reached at a median follow-up period of 12.4 months with osimertinib, while PFS was 13.9 months with standard first-generation EGFR TKIs (hazard ratio, 0.48; *p* = 0.14). CNS objective response rates were 91% and 68% in patients with ≥ one measurable CNS lesion (odds ratio, 4.6; 95% CI, 0.9 to 34.9; *p* = 0.66), and 66% and 43% in patients with one measurable and/or non-measurable CNS lesions (odds ratio, 2.5; 95% CI, 1.2 to 5.2; *p* = 0.11), for patients treated with osimertinib and first-generation EGFR TKIs, respectively [83]. In addition, based on analysis of predefined subgroups, the progression rate of intracranial disease was found to be lower in patients who were treated with osimertinib versus those who received alternative EGFR TKIs (6% versus 15%, respectively) [25]. Similarly, a longer mPFS was maintained in patients with CNS disease who received osimertinib versus those who received chemotherapy (11.7 vs. 5.6 months; *p* = 0.004) [84]. Interestingly, some evidence suggests an improved outcome in EGFR-mutated lung adenocarcinoma patients with brain metastases who receive initial radiation therapy (RT) [85]. Several studies are ongoing to evaluate whether the sequencing of treatment (RT followed by an EGFR TKI) versus deferring RT as a salvage treatment after initial use of an EGFR TKI; could improve outcomes.

## 3. Resistance Mechanisms in EGFR-Mutated Lung Adenocarcinoma That Compromise the Use of EGFR TKIs

**Figure 5:**
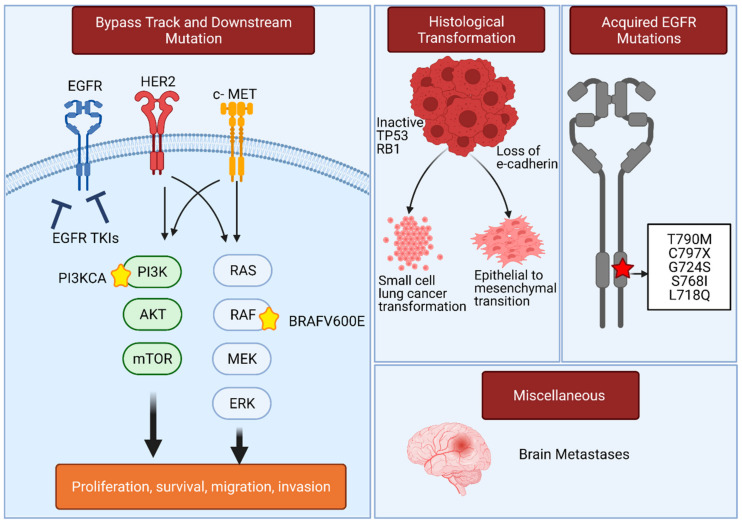
Common resistance mechanisms to EGFR TKIs in EGFR-mutated lung adenocarcinoma. Bypass track signaling include HER2 and c-MET amplification that can lead to parallel activation of downstream signaling, which is able to drive tumor proliferation despite EGFR inhibition. Downstream point mutations increasing activity of the EGFR effector kinases PI3KCA and BRAF^V600E^ can also lead to a constitutive activation of cancer growth. Histological transformation to SCLC, or epithelial to mesenchymal transition (EMT), contribute to loss of sensitivity to EGFR TKIs in lung adenocarcinoma. Acquired EGFR mutations in the tyrosine kinase domain can lead to increased affinity to ATP versus EGFR TKIs. Other mechanisms are also implicated in resistance to EGFR TKIs such as development of brain metastases.

Inherent and acquired resistance in EGFR-mutated lung adenocarcinoma pose a major challenge to outcome improvement in lung cancer treatment (Figure 5). The most frequent inherent resistant mechanism to EGFR TKIs is in-frame insertion of base pairs in exon 20 [86]. Acquired resistance can similarly be the result of novel mutations selected in EGFR that alter the ability of TKIs to bind or inhibit the protein. Alternatively, a change in cell phenotype to remove dependence on EGFR signaling, including upregulation of bypass receptor tyrosine kinases (RTKs), and histological transformation into SCLC or epithelial to mesenchymal transition, can cause resistance. Additionally, development of brain metastases has been proposed as a separate category of resistance [87]. Acquisition of a T790M gatekeeper mutation comprises about two thirds of acquired resistance developing during treatment with first- and second-generation EGFR TKIs and this issue has been largely resolved with the development of osimertinib, which has potent activity against T790M mutation. Novel mutations in EGFR that occur during treatment with osimertinib can contribute to disease progression and comprise approximately 6–10% of resistant mechanisms to osimertinib when it is used in a frontline therapy and 10–26% if osimertinib is used as a second line treatment [88].

Beyond the T790M mutation discussed above, one important mutation that contributes to acquired resistance to EGFR TKIs is the gatekeeper point mutation C797S. Occurring at a frequency of up to 14%, C797S prevents covalent binding of osimertinib to the ATP-binding pocket in EGFR, leading to therapy failure [89].

Establishment of parallel RTK signaling independent of EGFR (a bypass track) is another mechanism that contributes to resistance to TKIs. Among sources of EGFR bypass, *MET* gene amplification, causing increased activation of the MET kinase, is the most common cause of resistance to early generation EGFR TKIs and osimertinib (at frequencies of 5% and 15%, respectively) [45,88]. Similarly, amplification of the EGFR paralog ERBB2/HER2 can act as another bypassing oncogenic driver during treatment with EGFR TKIs. The prevalence of HER2 amplifications is higher in EGFR-mutated lung adenocarcinoma treated with early generation EGFR TKIs compared to treatment with osimertinib (5% versus 1–2% respectively) [88,89]. Other bypass track mutations that have been identified with a lower frequency in case reports include fusions in RET, ALK, and other genes that could act as oncogenic drivers [89]. Emergence of new mutations downstream of EGFR has also been described recently to confer resistance to TKIs and include *PI3KCA* and *BRAF^V600E^* mutations [89]. Preclinical studies have also demonstrated intersection between the VEGFR and EGFR pathways in tumor cells, and shown that the activating ligand for vascular endothelial growth factor receptor (VEGFR), VEGF, is sometimes overexpressed in resistant EGFR-mutated lung adenocarcinoma, providing autocrine signaling [90,91].

Phenotypic histological transformation to SCLC represents another major resistance mechanism in EGFR-mutated lung adenocarcinoma [92]. Acquired resistance through histological transformation accounts for up to 15% of resistant mechanisms to EGFR TKIs [89]. Management of transformed SCLC from EGFR-mutated lung adenocarcinoma is based on use of chemotherapy including etoposide/carboplatin and taxanes, which results in a median OS of 10.9 months [93].

## 4. Novel Treatment Approaches in EGFR-Mutated Lung Adenocarcinoma 

Development of novel therapeutics to overcome intrinsic and acquired resistance to EGFR TKIs is an area of extensive effort. Concurrently, ongoing investigations seek to determine whether combination therapies, such as addition of chemotherapy, VEGF/VEGFR2 monoclonal antibodies, and immune checkpoint inhibitors (ICIs) to use with EGFR TKIs could improve treatment outcomes.

### 4.1. Combining Chemotherapy and EGFR TKIs

Preclinical evidence of synergy between chemotherapy and EGFR TKIs in EGFR-mutated lung adenocarcinoma cell models raised the interest in assessing clinical efficacy in patients with EGFR-mutated lung adenocarcinoma [94]. For example, combination treatment with the folate inhibitor pemetrexed delayed the development of resistance to gefitinib in EGFR-mutated lung adenocarcinoma cell lines that had sensitizing exon 19 deletion mutations [95]. A phase II randomized trial compared the use of gefitinib versus gefitinib and pemetrexed in patients with EGFR-mutated lung adenocarcinoma. This study demonstrated prolonged mPFS in patients who received combination therapy, but a similar ORR and higher toxicity profile associated with combination treatment [96]. Interestingly, addition of pemetrexed and carboplatin to gefitinib in patients with EGFR-mutated lung adenocarcinoma showed statistically significantly higher ORR and improved OS in two phase III randomized clinical trials over gefitinib alone (75–84% versus 63–67%, respectively) and OS (50.9–not reached versus 17–38.8 months, respectively) [97,98]. Despite these promising results, the clinical relevance of such studies was impacted by the emergence of osimertinib as first line standard of care. The absence of direct comparison of these combinations with osimertinib and the unblinded design of these trials currently limits clinical applicability of such approaches. An important trial (FLAURA2) is currently underway to investigate the clinical efficacy of the addition of pemetrexed and carboplatin to osimertinib in patients with EGFR-mutated lung adenocarcinoma (NCT04035486, ClinicalTrials.gov).

### 4.2. Combining VEGF/VEGFR2-Directed Monoclonal Antibodies and EGFR TKIs

VEGF inhibitors inhibited cell growth of EGFR-mutated lung adenocarcinoma in preclinical studies suggesting that simultaneous targeting of both the EGFR and VEGFR signaling pathways could provide clinical advantage [99]. Clinical evidence of an added benefit of combined VEGF and EGFR inhibition is emerging. The phase II clinical trial JO25567 demonstrated a significantly prolonged mPFS in EGFR-mutated lung adenocarcinoma patients who received combination therapy (the VEGF inhibitor bevacizumab and erlotinib) versus those who were treated with single agent EGFR TKI (16 vs. 9.7 months, *p* = 0.0015) [100]. In contrast, another phase II trial using a similar comparative regimen did not find significant difference of mPFS between in patients treated with the combination [101]. A subsequent phase III randomized clinical trials demonstrated a longer median PFS in patients who received erlotinib and bevacizumab versus patients who received erlotinib monotherapy (16.9 vs. 13.3, *p* = 0.016); however, final OS analysis did not demonstrate a benefit with the addition of bevacizumab [102,103]. In addition, a recent phase II randomized clinical trial failed to demonstrate improved median PFS in patients with EGFR-mutated lung adenocarcinoma harboring T790M who progressed on prior early generation EGFR TKIs and were randomized to either osimertinib versus osimertinib plus bevacizumab [104].

Taking another approach to targeting VEGF signaling, the RELAY study was a phase III randomized clinical trial that evaluated the addition of the VEGFR2-directed monoclonal antibody, ramucirumab, to erlotinib in EGFR-mutated lung adenocarcinoma patients with defined EGFR TKI sensitizing mutations [105]. This trial showed significantly prolonged median PFS in patients who received erlotinib and ramucirumab versus patients who received erlotinib monotherapy (19.4 vs. 12.4, *p* < 0.0001) [105]. Additional ongoing studies are assessing the efficacy of adding bevacizumab and ramucirumab to osimertinib in frontline therapy (NCT04181060 and NCT03909334, ClinicalTrials.gov).

### 4.3. Immunotherapy and Chemo-Immunotherapy in EGFR-Mutated Lung Adenocarcinoma

Immune checkpoint inhibitors (ICIs) play a transformative central role in the treatment of lung cancer, significantly prolonging OS. The safety and efficacy of ICI in EGFR-mutated lung adenocarcinoma has hence been of considerable interest [106,107,108]. However, initial studies revealed low response rates to ICIs in patients with EGFR-mutated lung adenocarcinoma who received prior EGFR TKIs [109]. A phase II trial assessed the use of the programmed death-1 (PD-1) targeting antibody pembrolizumab as front-line therapy in EGFR-mutated lung adenocarcinoma patients with programmed death ligand-1 (PD-L1) expression >50%; there were 0 responses in 10 assessed patients, and one documented death due to pneumonitis within 6 months of treatment initiation [110]. A meta-analysis of several studies concluded that ICIs with either the PD-1 targeting agents pembrolizumab or nivolumab, or the PD-L1/2-targeting agent atezolizumab were not effective as second line treatment in patients with EGFR-mutated lung adenocarcinoma patients who progressed on prior EGFR TKI treatment [111]. Moreover, the high toxicity observed from various studies that evaluated either combination ICI and EGFR TKIs, or their sequential administration, has decreased the interest in further evaluation of this approach [112].

Of importance, a phase III randomized clinical trial IMpower150 evaluated efficacy of combination of atezolizumab with bevacizumab and chemotherapy in the treatment of lung cancer [113]. This trial included a subset of patients who had EGFR-mutated lung adenocarcinoma and demonstrated longer median PFS in those who received the experimental combination (atezolizumab, bevacizumab, and chemotherapy) compared to patients who received bevacizumab and chemotherapy alone [113]. A follow up exploratory analysis—the IMpower150 trial—analyzed the efficacy of the combination regimen in patients with EGFR-mutated lung adenocarcinoma, most of whom had sensitizing mutations, but who had progressed on earlier treatment with EGFR-targeting TKIs. This trial demonstrated an ORR of 71% and longer median PFS and median OS (hazard ratio of 0.41 and 0.31, respectively) [114]. The results of Impower150 have reinvigorated the idea that ICIs could have a potential role in the subset of lung cancer patients with EGFR mutations; however, much further study is needed.

### 4.4. Novel Treatment Strategies for Lung Cancer with EGFR Exon 20 Insertions 

Exon 20 insertions comprise approximately 3% of all lung cancer cases and up to 12% of EGFR-mutated lung adenocarcinoma [115]. As discussed above, currently available EGFR TKIs are thought to have very limited efficacy against exon 20 insertions (median PFS of 2 months) and chemotherapy remains the standard of care in these patients [116]; prognosis for patients treated with chemotherapy is inferior compared to that for patients with lung cancers bearing other sensitizing mutations in EGFR [117]. However, there may be some settings in which EGFR TKIs are effective in these patients. While two studies of osimertinib reported a low ORR of 5% in 17 lung cancer patients with exon 20 insertion mutations [118,119], one study showed a higher ORR (25%); this last study used a higher dose of osimertinib (160 mg versus the typical dose of 80 mg), and achieved some responses in patients who had progressed on prior therapy [76].

Mobocertinib (TAK-788) is a potent TKI developed to inhibit EGFR and ERBB2/HER2 [120]. A phase I/II single arm clinical trial evaluated mobocertinib in 27 patients with exon 20 insertions who had progressed on prior treatments; more than a third of these patients had brain metastases [121]. While about a third of patients had grade ≥3 diarrhea, a high ORR of 43% was observed, and a median PFS of 7.3 months. These results led to FDA breakthrough designation of mobocertinib for the treatment of EGFR-mutated lung adenocarcinoma with exon 20 insertions [121]. Currently, a phase III randomized clinical trial is underway to evaluate the safety and efficacy of mobocertinib versus chemotherapy as a front-line therapy for lung cancer patients with exon 20 insertions (NCT04129502). 

Amivantamab is an intravenously delivered bispecific antibody against EGFR and cMET [122,123]. The advantage of this novel monoclonal antibody includes its multi-mechanistic activity in lung cancer which involves targeting both EGFR and cMET signaling as well as its ability to induce antibody-dependent cytotoxicity in cancer cells. The activity of amivantamab against lung cancer with exon 20 insertion was demonstrated in phase I single arm study of 39 patients [124]. This study demonstrated an ORR of 36% and median PFS of 8.6 months with only 6% of grade >3 therapy related adverse events (TRAEs), which has resulted in FDA breakthrough designation for patients with exon 20 insertions [124].

Poziotinib is another novel TKI targeting EGFR, HER2/ERBB2, and ERBB4. In the phase II clinical trial ZENITH-20-1, 115 patients with lung cancer with exon 20 insertions who had received at least one prior line of therapy received 16 mg of poziotinib daily to investigate its safety and efficacy [125]. Results of this trial demonstrated an ORR of 19.3% and a median PFS of 4.2 months; however, more than half of the patients developing grade ≥3 TRAE [125]. These TRAEs required permanent discontinuation of poziotinib in 10% of the study population, and drug interruption in 88% of patients; 68% required dose modification during treatment. Given this high toxicity, subsequent trials are evaluating lower doses of poziotinib.

Finally, CLN-081 is another promising small molecule EGFR TKI that has been developed to target exon 20 insertions in lung cancer. In a phase I/IIa trial evaluating its safety and efficacy, the drug had good tolerability, and two out of five patients had partial response and three had stable disease after treatment initiation [126]. Notably, most of these patients had previously received either poziotinib or mobocertenib [126].

### 4.5. Novel TKI and Monoclonal Antibody Treatments Targeting Osimertinib Resistance Mechanisms

Despite the excellent efficacy of osimertinib in treating metastatic EGFR-mutated lung adenocarcinoma, the inevitable disease progression remains a pressing issue limiting further improvement of patient care. The combined use of gefitinib with osimertinib has been described in a case report of a patient with both T790M and C797S mutations [127]. In this case, there was rapid clearance of the C797S subclone despite continued progression of the disease, which suggests that tumors with C797S mutations could be sensitive to early generation EGFR TKIs. Similarly, another report demonstrated a response to erlotinib in a patient with EGFR-mutated lung adenocarcinoma who was initially treated with osimertinib and later progressed due to an acquired C797S mutation [128]. These results suggest the need for further investigation to evaluate the efficacy of combining or sequencing early generation EGFR TKIs with osimertinib in treatment of patients who develop C797S mutations. Another potential approach to treatment of C797X mutations may be to use novel allosteric EGFR inhibitors (EAI045 and JBJ-04-125-02), which are currently being investigated in preclinical studies [129,130]. Additionally, targeting gene fusions in combination with osimertinib in EGFR-mutated lung adenocarcinoma patients with either ALK-positive or RET-positive fusions was suggested to confer higher efficacy in this subset of patients, and warrants further validation in larger cohorts [131,132].

Given that enhanced MET signaling provides a significant source of resistance to osimertinib, targeting of this signaling pathway represents a promising approach. As mentioned previously, amivantamab is a bispecific antibody against EGFR and cMET that has shown activity and is currently being investigated in EGFR-mutated lung adenocarcinoma [124]. The TATTON study is a multi-center phase 1b open label trial that investigated the safety and efficacy of a potent selective c-MET inhibitor (savolitinib) plus osimertinib in lung adenocarcinoma patients who had MET amplification upon progression to EGFR TKI [133]. Combination therapy demonstrated an acceptable safety profile and high ORR reaching 64% in patients who received savolitinib. A phase II trial (SAVANNAH) is underway to evaluate the safety and efficacy of a savolitinib and osimertinib combination in EGFR-mutated lung adenocarcinoma patients who progress on osimertinib with detected MET amplification (NCT03778229, ClinicalTrials.gov).

Another important phase II clinical trial (ORCHARD) currently underway follows a unique design to evaluate the efficacy, safety, and tolerability of several novel therapeutics and combination treatments based on resistant mechanisms detected at time of progression on osimertinib (NCT03944772, ClinicalTrials.gov). Patients will receive different treatments including: osimertinib and savolitinib (for patients with MET amplification detected at progression to osimertinib); osimertinib and gefitinib (patients with EGFR C797X mutations); osimertinib and the EGFR targeting antibody necitumumab (patients with EGFR amplification); while another arm of patients who do not have a targetable biomarker will receive treatments such as ICI, chemotherapy, and osimertinib plus necitumumab [134].

Recently, overexpression of a surface membrane protein kinase receptor, AXL, was found to confer resistance to EGFR TKIs and to be associated with worse survival in lung cancer patients [135,136,137]. Currently, the inhibition of AXL tyrosine kinase receptor is being investigated as monotherapy or in combination with an EGFR TKI in lung cancer (NCT03255083 and NCT02729298, ClinicalTrials.gov). Finally, other novel EGFR TKIs are currently being evaluated for their safety and efficacy. For example, lazertinib (a novel third generation EGFR TKI) has demonstrated acceptable safety and high ORR (54%) in a recent phase I/II trial in patients with EGFR mutations (including T790M) who had progressed on early generation EGFR TKIs [138].

## 5. EGFR TKIs as Adjuvant Treatment in Locally Advanced EGFR-Mutated Lung Adenocarcinoma

The concept of introducing systemic cancer treatment in locally advanced lung cancer and other cancer types as adjuvant treatment has gained momentum in the last decade. Several clinical trials demonstrated improved outcomes (in terms of either PFS or disease-free survival used as primary endpoints) after the treatment of patients with ICIs in lung cancer, or BRAF-MEK inhibitors in locally advanced BRAF-mutated melanoma [139,140]. To this end, the prevalence of EGFR mutations in stage II and stage III lung adenocarcinoma is high (approximately 12%) [141]. The first phase III randomized trial to evaluate the safety and efficacy of EGFR TKIs as adjuvant therapy in EGFR-mutated lung adenocarcinoma was ADJUVANT/CTONG 1104 [142]. This trial evaluated gefitinib versus chemotherapy in resected EGFR-mutated lung adenocarcinoma, and demonstrated significantly prolonged disease-free survival in patients who received gefitinib. However, final OS analysis showed no significant benefit with geftinib versus chemotherapy combination (75.5 vs. 62.8 months, respectively) [143].

Using a similar approach, the phase III randomized ADAURA trial evaluated clinical outcomes in patients with EGFR-mutated lung adenocarcinoma (Stage II-IIIA) who received osimertinib for three years after surgical resection [144]. At 24 months, the disease-free survival was 90% in the osimertinib group versus 44% in the placebo group (*p* < 0.001), which has led to the 2020 FDA approval of osimertinib for the adjuvant treatment of resectable EGFR-mutated lung adenocarcinoma. Interestingly, the trial demonstrated a decrease in both locoregional recurrence and distant metastases in the osimertinib arm compared to the placebo arm (7% and 4%, versus 18% and 28%, respectively); final OS analysis is awaited [144]. Notably, there was 82% risk reduction for brain metastases recurrence or death in the osimertinib-treated group. Currently, a phase III trial (NeoADAURA) is ongoing for the evaluation of osimertinib plus chemotherapy as a neoadjuvant approach prior to surgery in patients with locally advanced EGFR-mutated lung adenocarcinoma (NCT04351555, ClinicalTrials.gov).

## 6. Conclusions

The emergence of a broad class of potent EGFR TKIs has revolutionized the management of EGFR-mutated lung adenocarcinoma. The progress of the landscape of EGFR TKI therapeutics in lung cancer from early generation to osimertinib and beyond reflects the depth of knowledge achieved in the last decade. The most common prevalent sensitizing mutations, including exon 19 deletions and L858R point mutations are susceptible to all generations of EGFR TKIs. Given the significant results of the FLAURA trial and the emergence of T790M mutations during treatment with early generation EGFR TKI, osimertinib is considered the standard of care as a frontline therapy in EGFR-mutated lung adenocarcinoma patients in the US and EU, and development of other new TKIs capable of inhibiting common resistance mutations is of high interest. Overall, acquired resistance remains a compelling challenge in the research and clinical field. To this end, recent trials and the availability of novel therapeutics and combination treatments have the potential to overcome resistance and improve outcomes in patients, particularly those with less common EGFR mutations. Finally, the recent implementation of EGFR TKIs in multimodality treatment of locally advanced EGFR-mutated lung adenocarcinoma represents a new precision medicine approach which promises to improve outcomes in these patients. 

## Figures and Tables

**Table 1:** Clinical Trials of EGFR TKIs in EGFR-mutated Lung Adenocarcinoma and Reported Outcomes.

Clinical Trial	Study Design	Patient Characteristic Highlights	ORR	mPFS (Months)	mOS	References
OPTIMAL	Erlotinib versus platinum-based doublet chemotherapy	Included: Presence of Exon 19del, Exon 21 missense mutation L858R Excluded: presence of brain metastases	83% vs. 36% *p* < 0.0001	13.1 vs. 4.6, *p* < 0.0001	22.8 vs. 27.2 *p* = 0.2663	[11,12]
EURTAC	Erlotinib versus platinum-based doublet chemotherapy	Included: Asymptomatic stable brain metastases Presence of Exon 19del, Exon 21 L858R missense mutation	64% vs. 18%	9.7 vs. 5.2 *p* < 0.0001	19.3 vs. 19.5 *p* = 0.87	[13]
ENSURE	Erlotinib versus platinum-based doublet chemotherapy	Included: Presence of Exon 19del, Exon 21 missense L858R mutation Excluded: Presence of brain metastases	62.7% vs. 33.6%	11 vs. 5.5 *p* < 0.0001	26.3 vs. 25.5 *p* = 0.607	[14]
WJTOG3405	Gefitinib versus platinum-based doublet chemotherapy	Included: Presence of Exon 19del, Exon 21 missense L858R mutation Excluded: Presence of symptomatic brain metastases	62.1% versus 32.2% *p* < 0.0001	9.2 vs. 6.3 *p* < 0.0001	34.9 vs. 37.3 *p* = 0.2070	[16,17]
NEJ002	Gefitinib versus platinum-based doublet chemotherapy	Included: Presence of Exon 19del, Exon 21 missense L858R mutation Excluded: Symptomatic brain metastases Presence of T790M missense mutation	73.7% vs. 30.7% *p* < 0.001	10.8 vs. 5.4 *p* < 0.001	30.5 vs. 23.6 *p* = 0.31	[15]
IPASS	Gefitinib versus platinum-based doublet chemotherapy	N/A	43% vs. 32.2% *p* < 0.001	N/A	18.8 vs. 17.4 *p* = 0.109	[18,19]
LUX-Lung 3	Afatinib versus platinum-based doublet chemotherapy	Included: Presence of Exon 19del, Exon 21 missense L858R mutation or other mutations	56% vs. 23% *p* = 0.001	11.1 vs. 6.9 *p* = 0.001	16.6 vs. 14.8 *p* = 0.60	[20]
LUX-Lung 6	Afatinib versus platinum-based doublet chemotherapy	Included: Presence of Exon 19del, Exon 21 missense L858R mutation or other mutations including T790M Excluded: Active brain metastases	66.9% vs. 23% *p* < 0.0001	11 vs. 5.6 *p* < 0.0001	22.1 vs. 22.2 *p* = 0.76	[21]
LUX-Lung 7	Afatinib versus gefitinib	Included: Presence of Exon 19del, Exon 21 missense L858R mutation Excluded: Active brain metastases	70% vs. 56% *p* = 0.0083	11 vs. 10.9 *p* = 0.017	27.9 vs. 25 *p* = 0.33	[22]
ARCHER 1050	Dacomitinib versus gefitinib	Included: Presence of Exon 19del, Exon 21 missense L858R mutation including T790M Excluded: History of brain metastases	75% vs. 72% *p* = 0.4234	14.7 vs. 9.2 *p* < 0.0001	34.1 vs. 26.8 *p* = 0.044	[23,24]
FLAURA	Osimertinib versus erlotinib or gefitinib	Included: Presence of Exon 19del, Exon 21 missense L858R mutation or other mutations Presence of stable brain metastases	80% vs. 76% 0 = 0.24	18.9 vs. 10.2 *p* < 0.001	38.6 vs. 31.8 *p* = 0.046	[25,26]

Abbreviations: ORR: objective response rate, mPFS: median progression-free survival, mOS: median overall survival, vs: versus, N/A: not applicable.

**Table 2:** Efficacy of select studies with approved EGFR TKIs in lung cancer with uncommon EGFR mutations.

Mutation	Frequency among EGFR Mutations	First Generation TKI	Second Generation TKI	Third Generation TKI	References
**Exon 18 Mutations**					
G719X	1.5–3% [66]	(erlotinib or gefitinib) ORR 36.8% * mPFS 6.3 mo [67]	Afatinib ORR 77.8% mPFS 13.8 mo [68]	Osimertinib ORR 52.6% mPFS N/A [69]	[66,67,68,69]
Exon 19 Mutations					
Exon 19 Insertions	1% [70]	(erlotinib or gefitinib) ORR 40% NA [66]	Afatinib ORR (3 partial responses in 4 patients) [71]	NA	[66,70,71]
Exon 20 Mutations					
Exon 20 Insertions	9% [72]	(erlotinib or gefitinib) ORR 8–27% mPFS 2–2.5 mo [73,74]	Afatinib ORR 8.7% mPFS 2.7 mo [68] Dacomatinib ** [75]	Osimertinib ORR 25% mPFS 9.7 mo [76]	[68,72,73,74,75,76]
S768I	0.59% [77]	(erlotinib or gefitinib) ORR 42% NA [66]	Afatinib ORR 100% mPFS 14.7 mo [68]	Osimertinib ORR 37.5% NA [69]	[66,68,69,77]
Exon 21 Mutations					
L861Q	3% [78]	(erlotinib or gefitinib) ORR 39% NA [66]	Afatinib ORR 56.3% mPFS 8.2 mo [68]	Osimertinib ORR 77.8% NA [69]	[66,68,69,78]

* Higher response rate and longer mPFS were observed with complex G719X mutations coexisting with other EGFR mutations. ** Reported 1 partial response, 2 stable disease, and 2 progressive disease in 6 patients with exon 20 insertions. Abbreviations: NA: not available, ORR: objective response rate, mPFS: median progression-free survival.
